# Emotions surrounding friendships of adolescents with autism spectrum disorder in Japan: A qualitative interview study

**DOI:** 10.1371/journal.pone.0191538

**Published:** 2018-02-06

**Authors:** Motofumi Sumiya, Kazue Igarashi, Motohide Miyahara

**Affiliations:** 1 Division of Cerebral Integration, National Institute for Physiological Sciences, Okazaki, Aichi, Japan; 2 Department of Child Studies, Shirayuri College, Chofu, Tokyo, Japan; 3 School of Physical Education, Sport and Exercise Sciences, University of Otago, Dunedin, New Zealand; Cinvestav-Merida, MEXICO

## Abstract

Emotions are embedded in culture and play a pivotal role in making friends and interacting with peers. To support the social participation of students with autism spectrum disorders (ASD) it is essential to understand their emotional life in the context of ethnic and school cultures. We are particularly interested in how anxiety and loneliness are experienced in developing and maintaining friendships in the daily encounters of adolescents with ASD in the specific context of Japanese schools, because these emotions could serve either as facilitators or barriers to social interaction, depending on how individuals manage them. The present qualitative study investigated perceptions of emotions related to friendship in the everyday school life of 11 adolescents with ASD in Japan. Data were collected by means of semi-structured individual interviews, which revealed a wide range of motivations for socialization, limited future prospects to deepen friendships, robust self-awareness of one’s own social challenges, and conscious efforts to cope with these challenges. An inductive approach to data analysis resulted in four themes: social motivation, loneliness, anxiety, and distress. To our knowledge this is the first study to uncover the rich emotional life of adolescents with ASD in the context of their friendships in an Asian culture.

## Introduction

Friendship is important for everyone throughout life. Of all developmental periods, adolescence marks a primary period of forming intimate friendships in typically developing youth [1,2]. In contrast, young people with autism spectrum disorder (ASD), whose defining feature is social impairments (DSM-5) [3], seem to follow different developmental trajectories because of their social communication challenges [See 4 for review]. For instance, children and adolescents with ASD are known to have fewer friends than age-matched peers [4,5]. Below the surface level of number of friends lie psychosocial processes involving individual perceptions of self and friends, and motivation to make friends and deepen friendships. How do young people with ASD feel about friends and friendships? Do they feel loneliness when alone or even when they are surrounded by other people? Do they want to have more friends, or would they rather isolate themselves from people and be alone? Given their self-awareness of limited social relationships [6,7] and their difficulties in emotional connection with friends [8], do young people with ASD have negative feelings of loneliness? To answer these questions in adolescents with ASD, this qualitative interview study delves into emotions in the context of friendship, as there has been a dearth of research investigating these topics in Asian cultures to date.

Investigations into emotional contributions to friendship have implications for a successful school life [9], mental health, and wellbeing [10]. It is particularly important to explore feelings within friendships of adolescents with ASD who may face an increasingly complex social milieu and become more aware of their interpersonal difficulties [11]. Previous studies in Western countries have identified feelings such as anxiety and loneliness within friendships in children and adolescents with ASD in the U.S.A. [12], Australia [13], and Israel [14]. Furthermore, Calder et al. [15] explored the desire for social relationships in children with ASD in the UK. In a study in the Netherlands, it was found that during adolescence, people with ASD experience an increase in social motivation and loneliness, which is positively correlated with social anxiety [16]. These findings in Western countries yield a picture of young people with ASD who have elevated motivation for social interaction as often failing in their social interaction with their peers and thus experiencing loneliness.

To date, the nature of friendship in young people with ASD has been explored by employing questionnaires [14,16,17], playground observation [5], and semi-structured interviews [1,18–22]. Among others, Carrington and her colleagues [18–20] conducted a series of studies that revealed how adolescents with ASD played with their friends, what sorts of difficulties they faced, and how they coped with these difficulties. Such descriptive data have been obtained in the U.S.A., UK, and Australia. To our knowledge, however, there has been a dearth of research that has documented in international academic literature the friendships of young people with ASD in the context of Asian cultures, using any of the above descriptive methods.

Before investigating the friendships of young people with ASD in an Asian culture, namely, Japan, in the present study, it is useful to establish the comparability of friendships between Eastern and Western cultures among non-ASD youngsters. Previous studies showed both similarities [23,24] and differences in young people’s friendships between the two cultures [25]. Similarities include the types of interactions with their friends, features of relationships such as reciprocity or prosocial behavior to the partner, and the importance of friends [23,24]. The differences involve particular aspects of friendship, such as the expected emotions gained from interactions with friends. For example, Japanese young people enjoy comfort and avoid uncertainty in their friendships more than surprise and excitement, which their American counterparts enjoy [25]. Japanese people also prioritize social harmony in interpersonal relationships and value peer acceptance more than people in Western societies do [25–27]. Thus, there are some unique cultural features of friendship in Japan, and culturally sensitive and responsive support and education have been proposed [28–31]. To formulate culturally specific strategies to support young people with ASD in Japan, it is essential to first explore their behavioral and emotional experiences, understanding, and motivation for friendship qualitatively in a specific cultural context.

Qualitative methods can provide a deeper understanding of friendship in ASD from the perspective of adolescents with ASD themselves [32–33], and highlight the roles that anxiety and loneliness play in developing and maintaining friendship in the daily encounters of adolescents with ASD. Qualitative methods can also empower voices of adolescents with ASD, who often go unheard in autism research [34]. Furthermore, qualitative research gives important information to teachers and clinical service providers [32]. Therefore, qualitative methods are highly beneficial for understanding how adolescents with ASD themselves construct the social world of school [35] in the Japanese school system.

To bridge the previously mentioned research gaps, our study aims to 1) describe friendships in adolescents with ASD in Japan, and 2) explore the experience of anxiety and loneliness in the context of the desire of adolescents with ASD to make friends at school.

## Methods

### Participants

A total of 11 high-functioning adolescents with ASD (8 males (73%) and 3 females (27%)) whose verbal IQ on WISC-III was over 85, aged 10 to 15 years were recruited from private remedial centers, college-based remedial teaching centers, and special education classes in Tokyo and Okayama, Japan. They were all students from different elementary or junior high schools. Two of the 11 participants (18%) were diagnosed with Autistic disorder (AD), seven with Asperger’s disorder (AS) (63%), and two with Pervasive Developmental Disorder (PDD) (18%). Because Shirayuri College had neither a specific institutional review board nor an ethics committee, the present study was reviewed and approved by a one-off committee, named the ‘Provisional Ethics Committee’, consisting of a group of developmental psychologists in the college, and also by the heads of the centers and schools from which students were recruited. Participation in this study was voluntary, and prior to the interview, all students with ASD and their parents signed informed consent forms according to the principles in the Declaration of Helsinki. Prior to the interview, the interviewer orally explained to the participants that the interview concerned their friendships, that they did not have to answer any questions if they did not want to, and that there were no right or wrong answers to the interview questions.

To confirm the participants’ clinical manifestation and cognitive profile of ASD, the Japanese version of the Autism Spectrum Quotient (AQ) [36] was administered to their parents. The AQ score and demographic data of each participant are summarized in Table 1; furthermore, we have provided supplemental tables giving additional cognitive evidence consisting of the definitions of friendship (S1 Table) and loneliness (S2 Table) by each participant and those of same-age neurotypical adolescents whose data were collected in this study as an additional information source.

**Table 1:** Demographic information and clinical characteristics of the participants.

Name	Jiro	Ken	Anna	Akira
Sex	Male	Male	Female	Male
Grade	5th	5th	9th	9th
Age (years)	12	11	15	15
Diagnosis	AD	AD	AS	AS
Verbal IQ	110	87	92	90
AQ	23	36	24	44
Features	many fights in school	having difficulties thinking and focusing	knows her own difficulties	OCD, afraid of be scolded by teachers
Name	Michio	Haruo	Eigo	Shiho
Sex	Male	Male	Male	Female
Grade	7th	6th	6th	5th
Age (years)	13	12	12	11
Diagnosis	AS	AS	AS	AS
Verbal IQ	96	100	142	94
AQ	35	36	32	32
Features	depression		experience of bullying	ADHD, Learning Disorder, lies at home
Name	Rie	Toshi	Hiro	
Sex	Female	Male	Male	
Grade	7th	9th	5th	
Age (years)	13	15	12	
Diagnosis	AS	PDD	PDD	
Verbal IQ	85	110	100	
AQ	31	32	24	
Features	depression, self-harm	school refusal	Learning Disorder, difficulty coping with his irritability	

AD, autistic disorder; AS, Asperger’s disorder; PDD, Pervasive Developmental Disorder; OCD, Obsessive-Compulsive Disorder; AQ, autism spectrum quotient

To protect the identities of the participants and of people discussed in the interviews, we used pseudonyms and slightly altered the ages of the participants to an extent that would not affect the interpretation of the interview data.

### Data collection and analysis

We employed individual semi-structured interviews to discover the feelings of loneliness and anxiety related to friendship. All participants were Japanese students, as noted, and the interviews were conducted by the first author in the Japanese language at the participants’ schools, clinical institutions, or the participants’ homes. At the time of the interviews, the interviewer (MS) was a master’s student majoring in clinical psychology with three years of fieldwork experience with students with neurodevelopmental disorders. If the participating students, their parents, or their teachers requested or recommended the presence of parents or teachers during the interview, they were allowed to be present at the interview, with the student’s consent. Six of the 11 students asked for the presence of either a parent or a teacher in the interview. When the response of the interviewee was unclear, the interviewer asked the parent or the teacher to clarify the response during the interview. The remaining five students did not ask for adults to accompany them, and the interviewer secured these interviewees’ permission to ask their parents or teachers for clarification and confirmation after the interviews.

Before the interviews, we prepared questions concerning friendship (see supplemental information). We used the questions developed by Carrington, Templeton, and Papinczak [20], and added some of our original questions about loneliness (e.g., “When do you feel lonely?”) and anxiety (e.g., “When you are with your friends, how anxious are you?”). Furthermore, we decided to ask the participants who their friends were because a previous study by Bauminger and Kasari [17] showed that children with ASD had mentioned their teacher aids and parents as friends. All questions were made available in advance upon request. At the beginning of each interview, the interviewer established rapport with the interviewee by talking about the participant’s hobbies and what they enjoyed recently. Then the interviewer explained the purpose of the interview: “Some children are good at making friends, and other children are not so good at making friends. I am interested in how you interact with your friends.” When the interviewer actually asked the preplanned questions, the words were modified in accordance with each participant’s language ability. If participants did not seem to understand the questions, the interviewer rephrased them, trying to do so in accordance with the participant’s language ability, and provided prompts, while trying not to ask leading questions. When interviewees had difficulty communicating about their emotions, the interviewer focused on behavioral aspects of friendship. When the interview topic covered sensitive issues, such as feelings of loneliness or anxiety, the interviewer expressed to the students how much he appreciated their disclosure of feelings and experiences. After each interview, the interviewer ensured with each interviewee’s parent or teacher that the interviewee was not distressed by the interview.

A total of 20 to 30 questions were asked in each interview; interviews lasted from 20 to 40 minutes. All interviews were recorded with a digital audio-recorder and were transcribed verbatim. Thematic analysis [37] was used to examine and identify themes within the interviews, following these six steps: familiarizing data, generating codes, searching for themes, reviewing themes, defining and reviewing themes, and producing the report. The transcribed interview data were coded by the first author (MS) and two coders who were students pursuing their masters in clinical and developmental psychology (SM and YS), and the underlying themes were agreed upon following discussions across multiple meetings. The second (KI) and the third author (MM) served as methodological auditors to examine the credibility of the conceptual interpretation of the original data [38]. We used QDA software (Weft QDA, www.pressure.to/qda/) to analyze and organize the data.

## Results

Nine of the 11 students responded to the interview questions in detail, with specific examples. One participant took relatively long pauses before answering questions, and the remaining participant provided only brief answers. The interview data yielded four major themes: social motivation, loneliness, anxiety, and distress.

### Social motivation

Seven out of 11 students had at least one friend who we considered to be in a reciprocal friendship with them; two students were in a one-way friendship, whose reciprocity could not be confirmed; and the two remaining students had no friendships at all, according to the information first provided by the students and later confirmed by their parents and schoolteachers.

Some of the students who claimed to be in reciprocal friendships seemed to have a low level of motivation to interact with their friends. Around the time of the interview, Haruo only had one friend, whose name was Makoto. Haruo explained how he would spend time with his friend:

*Interviewer*: *What do you do when you play with your friend?*

*Haruo*: *I very rarely play games [on Nintendo DS]*.

*Interviewer*: *How do you spend time with your friends at school*? *What do you do with him?*

*Haruo*: *I do nothing with him at school*.

He told the interviewer that he did not hang out with anyone at school, and that he rarely played with Makoto even at home. The only game he played with Makoto was *Pokémon* on Nintendo DS. The range of activities through which Haruo interacted with his friend, Makoto, seemed to be rather limited. The interviewer asked Haruo what kind of help he needed from teachers to make friends or develop friendship, hoping to gain information for the teacher’s possible intervention. Haruo’s answer was “nothing.” In response to the interviewer’s question regarding the motivation to further develop friendship, Haruo answered “rather low” followed by “no prospects of future friendship.” Even though it was very rare for Haruo to hang out with Makoto, his motivation for further friendship was not high.

Similarly, Jiro also reported that his motivation to deepen friendship with his few friends was “rather low.”

*Interviewer*: *Do you think you need friends?*

*Jiro*: *Of course*, *I do*. *But even if I don’t have any friends at all*, *I wouldn’t be sad*. *That only means that I would get bored easily and that no one would care about me*.

Jiro needed friends who would occupy his time and care about him. He seemed to want a friend “in need,” and not a reciprocal friendship. Such a one-sided relationship would not develop into a deeper friendship, which Jiro might be well aware of:

*Interviewer*: *How much are you motivated to develop friendship?*

*Jiro*: *Not very much*.

*Interviewer*: *Do you know why you are not so motivated to develop friendship?*

*Jiro*: *Yes*, *that’s because I have enough friends already*. *I can’t say “Let’s be friends” for my own sake*. *Well*, *people become friends spontaneously*, *I think*.

Jiro was well aware of the standard ways of making friends. In his case, he anticipated challenges to deepening friendships. The interview with Jiro uncovered that his way of “interacting with friends” was limited to rough-and-tumble play, such as wrestling and sumo. In fact, Jiro mentioned that he had inflicted injuries on his friends a few times. As a result, Jiro usually played by himself because his invitations to wrestle declined. Jiro’s low motivation to develop friendship might be due to his learned helplessness from his narrow and rather dangerous repertoire of skills related to interaction with other students.

In contrast, some other participants were highly motivated to socialize, but for different reasons. For example, Michio wanted to make as many new friends as possible in his new class, whereas Eigo and Rie only wanted compatible friends, and the number of friends did not matter. Thus, some emphasized the quantity, and some the quality of friendships. Yet others, such as Shiho, valued both quantity and quality of friends; however, she had difficulty approaching her classmates.

*Shiho*: *It doesn’t matter how many friends I have*, *it is hard to make myself look friendly to my classmates*.

*Interviewer*: *Do you mean you don’t know how to be friendly to your classmates?*

*Shiho*: *People’s personalities are different*, *and their responses are also different when I approach them*. *So*, *I don’t know how to be friendly*.

Extracurricular activities can serve as vehicles for socialization. Of the 11 participants, three students took part in extracurricular activities (namely, basketball club, school choir, history club, and railway club). Michio expressed how he liked playing ball games:

*Interviewer*: *What do you enjoy doing with your friends?*

*Michio*: *Playing*, *playing outdoors*. *I belong to a school basketball club*, *so I like playing basketball*.

*Interviewer*: *What kind of outdoor activities do you do?*

*Michio*: *Naturally*, *I like playing basketball very much*, *and I also like playing dodgeball*.

In sum, the participants’ levels and types of social motivation to make friends varied, although most of them valued friendship highly. The students who had no friends generally wished to make friends. Thus, with a few exceptions, the adolescents with ASD were not satisfied with their own world, but appreciated and desired friendship.

### Loneliness

The theme of loneliness emerged, reflecting the feelings of adolescents with ASD who had difficulty casually socializing with their peers at school and in other group situations. There were a variety of situations in which participants experienced loneliness, such as situations where they were in groups but not surrounded by friends, and situations where bidirectional interactions were not occurring. By contrast, some participants reported that they had no experience of loneliness.

Rie goes to a special support school for students who are unable to cope with mainstream schools for various reasons. Rie considers Lisa, Tomoko, and her homeroom teacher, Mr. Shirai, to be her friends because she can easily converse with them. In their absence, Rie has no one to talk to or spend time with.

*Interviewer*: *When do you feel lonely?*

*Rie*: *When Lisa*, *Tomoko*, *and Mr*. *Shirai are not around me*.

*Interviewer*: *Are they not often around you?*

*Rie*: *Mr*. *Shirai leaves there [special support school] in the middle of the day*, *and Lisa and Tomoko are absent basically [because they are skipping school]*.

*Interviewer*: *Do you often feel lonely at the special support school?*

*Rie*: *Yes*, *very often*.

Rie also mentioned that she felt lonelier when her friends talked with other students than when her friends were not around. It seems that Rie’s desire to monopolize her limited number of friends, coupled with her lack of options to interact with other students, exacerbated her loneliness.

Adolescents with ASD can feel loneliness even when they are interacting with their classmates in a group situation. Jiro explained how this happened.

*Jiro*: *When we were playing tag together*, *other students didn’t catch me and they didn’t let me catch any one of them*. *I felt loneliness then*.

It seems that Jiro was being selectively excluded as a target of ‘ijime’ which is the Japanese term for bullying with a less physically violent connotation and greater emphasis on social manipulation [39], probably because he had physically harmed others before. Given that children with ASD are thrice more likely to be victims of bullying than non-autistic children [40,41], Jiro might have been at a risk for bullying. His experience indicates that physical presence in a group is not enough; meaningful interactions are necessary for students with ASD to feel included in and connected with the group.

Although some participants could elaborate on both their understanding and experiences of loneliness, others understood loneliness only on the conceptual level without any experience of the feeling. For example, Shiho seemed to know what loneliness was; she explained her understanding as “sad for being alone, and nobody is around to talk with.” However, she said that she had not felt loneliness even when she had no friends.

### Anxiety

Adolescence is a remarkable period when the development of social cognition, which young people to tune into social differences [42,43]. This tendency of adolescents increases the risk of anxiety symptoms particularly in persons with ASD [44]. Whether it is specific to a particular topic of conversation or general to any kind of social situation, anxiety affects social interactions.

Conversation topics were an important theme for adolescents with ASD. Shiho confided that she was constantly worried whether she could keep up with conversations. In the case of Rie, her concern was whether the person she conversed with knew the topic she wanted to talk about.

Eigo and Toshi also had more general anxiety regarding friendship.

*Interviewer*: *Are you worried about something when you are with your friends?*

*Eigo*: *Yes*, *I am always worried whether they stay friends with me*.

*Interviewer*: *What is the reason for that*? *Did anything happen to you in the past which makes you worry about losing your friends?*

*Eigo*: *No*, *nothing really*.

In another case, Hiro was deeply worried if he could make new friends, because he wanted to have friends very much without knowing how to accomplish this. It seems that his high level of social motivation made him worried and anxious, a phenomenon identified by White, Bray, and Ollendick [44]. Such anxiety apparently impeded his chance of successfully making new friends.

### Distress

Distress in social situations interferes with the successful development of social relationships [45]. Carrington and Graham [18] referred to “masking,” which children with ASD use as a device to hide their negative feelings towards and difficulty in social interactions. Rie explained how she uses masking:

*Interviewer*: *Have you ever put on an act when you hung out with your friends?*

*Rie*: *Yes*, *I have*.

*Interviewer*: *How come you put on an act?*

*Rie*: *Because I wanted to be praised and favored*.

*Interviewer*: *Do you put on an act with everyone?*

*Rie*: *Yes*, *I do basically*.

…

*Interviewer*: *How does it make you feel?*

*Rie*: *It makes me feel tired*.

Rie said she would often pretend to understand conversations that she had trouble following. In addition to putting on an act, Rie said she let her friends win in card games to preserve friendship. Masking, or masquerading [18], may help with adaption to social situations, but excessive use may cause other types of distress. In Rie’s case, she grows weary after making so much social effort.

Masa was annoyed by his friends’ unpredictable behaviors, such as suddenly leaving a group or jumping into a group. This annoyance might reflect the difficulty young people with ASD experience predicting others’ behaviors [46]. Whatever the causes of distress, adolescents with ASD need to make extraordinary efforts to engage in social relationships and endure unavoidable social stress doing so.

## Discussion

Our interview data revealed different degrees of desire in these adolescents with ASD to socialize with peers, limited future prospects to deepen friendships, solid awareness of their own social limitations, and impressive efforts to cope with these limitations. We also uncovered the complex roles that feelings of anxiety and loneliness played in their process of maintaining friendships. Some participants were anxious to behave properly and afraid to lose friends. Others felt lonely when they felt rejected or excluded. Yet others claimed that they had limited feelings as such. In the following discussion, we describe friendships in adolescents with ASD in Japan and contrast them with those in other cultures, and explore the experience of anxiety and loneliness in the context of the desire of adolescents with ASD to make friends at school.

### Possible uniqueness of coping strategies in Japanese adolescents with ASD

There seem to be similarities and differences between the Japanese participants in the current study and individuals with ASD in Western cultures reported on in previous studies on masking coping strategies to maintain friendships [19–20,22]. Among the Japanese participants who felt rejected and excluded were adolescents who masked their feelings, as also seen in the case of Australian adolescents with ASD [19,20]. In the study of Carrington et al. [19], Australian participants with ASD confided that they concealed their social awkwardness by behaving in the same ways as others and pretending to share the same interests. Likewise, Rie would often pretend to understand conversations that she had trouble following. Even though acting in this way was stressful to her, she used this strategy to avoid peer rejection. This common masking strategy may be based on the knowledge that sharing interests and participating in common activities are important for friendship [21].

A possible cultural difference between Japan and Australia in masking coping strategies may be seen in coping strategies. Rie used an internal coping technique and sacrificed herself by masking her desire to win and intentionally losing in the card game. This coping technique may be unique to Japanese culture, which emphasizes social harmony over self-interest [25–27]. In contrast, an Australian adolescent with ASD employed an external coping technique by pretending to have a wide social network, such as a fictional account of an extensive list of friends and fabricated stories about interactions with them [20]. The opposite directions of these coping strategies may reflect differences between Eastern and Western cultures in that 1) people in Eastern cultures tend to set coping goals that focus on the needs of others, relatedness, and interdependence, which may require self-sacrifice, whereas people in Western cultures tend to focus on their own needs, asserting independence and control of the external environment [47]. These cultural differences in the direction of coping strategies might necessitate different directions in the masking strategies used by adolescents with ASD to adapt to social situations. If internalizing coping is unique to Japanese individuals among people with ASD, parents and teachers should be aware of this tendency and teach culturally appropriate social skills [31]. However, further study is needed to confirm if this unique coping tendency apparent in this study actually holds true.

### Emotions surrounding adolescents with ASD

The levels and types of friendship-related social motives and emotional experiences varied among the students with ASD who participated in this study. Consistent with a previous study by Chen et al. [48], a high level of social motivation made some individuals with ASD, such as Hiro, worried and anxious about friendship without knowing how to make friends. Such anxiety could either keep them motivated, or, worse, impede their chance of successfully making new friends. More studies are needed to explore how social anxiety relates to maintaining and developing friendships in their daily school life.

In another case, Jiro, whose only way of hanging out with his friends was rough-and-tumble play, such as wrestling and sumo, seemed to be ostracized in his classroom. Jiro reported that he had inflicted injuries on his friends a few times during such play. Thus, he might be socially excluded by his classmates (e.g., when nobody tried to catch him during play tag). The social demands around him might have exceeded the capacity of his social skills to cope [49]. According to an experimental study [50], adolescents with ASD are sensitive to ostracism and experience increased anxiety and need threat following it. Therefore, Jiro’s loneliness and low motivation to develop friendships might have followed from the situations he experienced during play.

There were students with ASD who reported they had limited feelings. There are two possibilities: one is that they actually feel nothing, while the other is that they have feelings but have difficulties recognizing and explaining their own feelings and those of others [51,52]. Indeed, some researchers [53,54] pointed out that individuals with ASD have emotional interoception difficulties similar to those in individuals with alexithymia. Recently, Bird and Cook [55] proposed that deficits in recognition of emotions or the internal state of the body are associated with co-occurring alexithymia for some individuals with ASD, although alexithymia is not commonly considered characteristic of ASD [56]. Alternatively, as suggested, there may be a genuine lack of certain emotions for some participants; whichever is the case, this study has extended the growing knowledge on the friendships of adolescents with ASD by focusing on their emotional life, particularly their feelings of loneliness and anxiety.

### Limitations

Our study has shed light on the emotional life of students with ASD in the context of their friendships, but the limitations of this study need to be considered for the interpretation of the findings and for future research. The participants were few in number, and all of them were clinically diagnosed with autism disorder, Asperger’s syndrome, or pervasive developmental disorder according to DSM-IV-TR [57] by medical doctors specialized in child psychiatry or developmental pediatrics. Future studies need to use contemporary diagnostic criteria of ASD and its comorbid conditions defined in DSM-5 [3] and gold-standard measures such as the Autism Diagnostic Interview-Revised (ADI-R) [58] or the Social Communication Questionnaire [59], which were not available in Japanese at the time of this research (2011), for the verification of the ASD diagnosis.

A recent study by Dean et al. [60] found that children with ASD preferred to socialize with same-gender friends, as did their typically developing counterparts. Future research could yield different dimensions of insight into these friendships. For example, this line of studies needs to tap into the emotional life of individuals with ASD in different age groups, such as young children and adults, because the nature of friendship is likely to change throughout life.

### Conclusion

This study is probably the first to uncover the rich emotional life of adolescents with ASD in the context of their friendships in an Asian culture. In addition to emotions such as anxiety, loneliness, and desires for social relationships that have already been identified by previous studies conducted in Western cultures, we discovered a possibly unique coping strategy characterized by internalization and self-sacrifice in our study.

## Supporting information

S1 File Questions after Carrington et al., 2003 and original questions. (DOCX)

S1 Table Definitions of friendship. (DOCX)

S2 Table Definitions of loneliness. (DOCX)
