# Supplementary material for: Emotions surrounding friendships of adolescents with autism spectrum disorder in Japan: A qualitative interview study
Source: PLoS One. 2018 Feb 6;13(2):e0191538. doi: 10.1371/journal.pone.0191538 (PMC5800535; doi:10.1371/journal.pone.0191538)
Supplement: S1 File — (DOCX) [file pone.0191538.s001.docx]

**Supplemental information**

Questions after Carrington et al., 2003

- Tell me what kind of people you consider as friends.
- Tell me what kind of people you consider as acquaintances.
- Tell me what kind of people are not your friends.
- What are the differences between friends and best friends?
- Do you have some friends? Do you have best friends?
- Who are your friends? Do you interact with them at school, around home, or somewhere else?
- Are your friends younger or older than you? Tell me about these people. How are they different from you? What are the similarities between you and them?
- What sort of things do you like to do with your friends?
- What sort of things do you do when you are with other people of your age?
- Sometimes things with friends go well. Tell me about these things.
- Sometimes things with friends do not go so well. Tell me about these things.
- Is there anyone who is no longer your friend? If so, what were the things that didn’t work well between you and him or her?
- What sorts of things are difficult with your friends and getting along with other kids?
- What do you enjoy with your friendship and what is working well in your friendship?
- Do you have any other things which you enjoy in your friendship?
- What is important in your friendship?
- In the future how would you like your friends to be?
- What are the things that you don’t want to change in your friendship?
- What are the things that you like to change in your friendship?
- Do you want your friends to know you better? If so, what do you want them to know about you? What do they need to know about you?
- What shouldn’t your friends know about you?
- Tell me how you want to be helped for your friendships.
- What kind of help would you need from your teachers?
- What other kinds of help would you need?

Original questions

- When you hang out with your friends, have you pretended to be someone else?
- Have you been misunderstood by good friends? Please tell me your experiences. Are there some things that are misunderstood or cannot be understood by your friends?
- When do you feel loneliness?
- When do you think people feel lonely?
- Do you think some people can be lonely among a group of people?
- Do you think some people can feel lonely even when they are with good friends?
- Do you need friends in your school life?
- Do you want to develop your friendship further? (Do you want more friends? Do you want to deepen your friendship with the friends you have now? Like, you want more friends, or you want to be closer to friends you have right now.)
- When do you feel happy, when you are playing by yourself or when you are with your friends? Like, when you are with your friends, or playing by yourself, or studying?
- When you are with your friends, how anxious are you?
