# Supplementary material for: Emotions surrounding friendships of adolescents with autism spectrum disorder in Japan: A qualitative interview study
Source: PLoS One. 2018 Feb 6;13(2):e0191538. doi: 10.1371/journal.pone.0191538 (PMC5800535; doi:10.1371/journal.pone.0191538)
Supplement: S1 Table — (DOCX) [file pone.0191538.s002.docx]

**Supplemental table 1. Definitions of friendship**

| ASD group | | | | TD group | | | |
| --- | --- | --- | --- | --- | --- | --- | --- |
| Name | Age | Gender | Definitions | Name | Age | Gender | Definitions |
| Jiro | 12 | Male | People in good relationships | Daichi | 11 | Male | People you get along with |
| Ken | 11 | Male | Companions | Hideo | 13 | Male | People you talk to in class and play with |
| Anna | 15 | Female | People who listen to your concerns that cause worry | Izumi | 15 | Female | People who care about you and always play with you |
| Akira | 15 | Male | People who have a common interest in trains | Naoki | 14 | Male | People you have fun and spend time with |
| Michio | 13 | Male | People who listen to each other and sort out problems together when in trouble, and those who muck around with you when playing | Taka | 14 | Male | Important people |
| Haruo | 12 | Male | People who stay around you wherever you are going | Yoshi | 14 | Male | People who are fun to be with |
| Eigo | 12 | Male | People who understand you and with whom you get along very well | Tomo | 13 | Male | People you play with |
| Shiho | 11 | Female | People who are on your side when you are having conflicts with others. People who understand you and give your support | Eiji | 11 | Male | People who help you when you have problems |
| Rie | 13 | Female | People you go out with and interact with privately | Jun | 12 | Female | People who are fun and kind |
| Toshi | 15 | Male | People who are fun to be with, like those with whom you laugh | Hiroko | 13 | Female | People who understand you |
| Hiro | 12 | Male | People who play together with everyone | Yosuke | 11 | Male | People who are easy to talk to |

ASD, autism spectrum disorder; TD, neurotypical development
