# Supplementary material for: Emotions surrounding friendships of adolescents with autism spectrum disorder in Japan: A qualitative interview study
Source: PLoS One. 2018 Feb 6;13(2):e0191538. doi: 10.1371/journal.pone.0191538 (PMC5800535; doi:10.1371/journal.pone.0191538)
Supplement: S2 Table — (DOCX) [file pone.0191538.s003.docx]

**Supplemental table 2. Definitions of loneliness**

| ASD group | | | | TD group | | | |
| --- | --- | --- | --- | --- | --- | --- | --- |
| Name | Age | Gender | Definitions | Name | Age | Gender | Definitions |
| Jiro | 12 | Male | When people are quiet | Daichi | 11 | Male | When someone is left out of the group |
| Ken | 11 | Male | (showed sad face as an answer) | Hideo | 13 | Male | When someone is left out of the group or bullied |
| Anna | 15 | Female | When you are excluded | Izumi | 15 | Female | When people are alone at home |
| Akira | 15 | Male | When you are left out and no one cares about you | Naoki | 14 | Male | When nobody pays attention to you |
| Michio | 13 | Male | If one person is hated and bullied by another person and abandoned by the others, then that person may feel lonely | Taka | 14 | Male | When people are bullied |
| Haruo | 12 | Male | I don’t know | Yoshi | 14 | Male | I don't know |
| Eigo | 12 | Male | Perhaps, when you are excluded, and possibly when you are alone | Tomo | 13 | Male | When people laugh at your mistakes, say you are dirty, and give a chuckle |
| Shiho | 11 | Female | When you are lonely and have no one to talk to | Eiji | 11 | Male | Someone who you thought was a friend betrays you |
| Rie | 13 | Female | When people are alone | Jun | 12 | Female | When people are left out of the group |
| Toshi | 15 | Male | When people are bullied | Hiroko | 13 | Female | I don't know |
| Hiro | 12 | Male | When people are left alone | Yosuke | 11 | Male | When people don't pay any attention to you |

ASD, autism spectrum disorder; TD, neurotypical development
